# Neuroprotective effects of quinpirole on lithium chloride pilocarpine-induced epilepsy in rats and its underlying mechanisms

**DOI:** 10.1186/s40001-024-01694-x

**Published:** 2024-02-14

**Authors:** Hui Wang, Yongheng Zhao, Dongqing Zhang, Jun Li, Kun Yang, Junli Yang, Baomin Li

**Affiliations:** 1 Department of Pediatrics, Qilu Hospital of Shandong University, Jinan, Shandong China; 2 Department of Pediatrics, The Affiliated Taian City Central Hospital of Qingdao University, Taian, Shandong China

**Keywords:** Epilepsy, Quinpirole, Neuroprotection, GluR2, BAX, BCL2

## Abstract

**Introduction:**

Epilepsy is a common neurological disorder that presents with challenging mechanisms and treatment strategies. This study investigated the neuroprotective effects of quinpirole on lithium chloride pilocarpine-induced epileptic rats and explored its potential mechanisms.

**Methods:**

Lithium chloride pilocarpine was used to induce an epileptic model in rats, and the effects of quinpirole on seizure symptoms and cognitive function were evaluated. The Racine scoring method, electroencephalography, and Morris water maze test were used to assess seizure severity and learning and memory functions in rats in the epileptic group. Additionally, immunohistochemistry and Western blot techniques were used to analyze the protein expression levels and morphological changes in glutamate receptor 2 (GluR2; GRIA2), BAX, and BCL2 in the hippocampi of rats in the epileptic group.

**Results:**

First, it was confirmed that the symptoms in rats in the epileptic group were consistent with features of epilepsy. Furthermore, these rats demonstrated decreased learning and memory function in the Morris water maze test. Additionally, gene and protein levels of GluR2 in the hippocampi of rats in the epileptic group were significantly reduced.

Quinpirole treatment significantly delayed seizure onset and decreased the mortality rate after the induction of a seizure. Furthermore, electroencephalography showed a significant decrease in the frequency of the spike waves. In the Morris water maze test, rats from the quinpirole treatment group demonstrated a shorter latency period to reach the platform and an increased number of crossings through the target quadrant. Network pharmacology analysis revealed a close association between quinpirole and GluR2 as well as its involvement in the cAMP signaling pathway, cocaine addiction, and dopaminergic synapses.

Furthermore, immunohistochemistry and Western blot analysis showed that quinpirole treatment resulted in a denser arrangement and a more regular morphology of the granule cells in the hippocampi of rats in the epileptic group. Additionally, quinpirole treatment decreased the protein expression of BAX and increased the protein expression of BCL2.

**Conclusion:**

The current study demonstrated that quinpirole exerted neuroprotective effects in the epileptic rat model induced by lithium chloride pilocarpine. Additionally, it was found that the treatment not only alleviated the rats' seizure symptoms, but also improved their learning and memory abilities. This improvement was linked to the modulation of protein expression levels of GLUR2, BAX, and BCL2. These findings provided clues that would be important for further investigation of the therapeutic potential of quinpirole and its underlying mechanisms for epilepsy treatment.

## Introduction

Epilepsy is a common neurological disorder characterized by recurrent seizures and cognitive impairments. However, significant challenges persist in understanding its underlying mechanisms and developing effective treatment strategies. Despite extensive research on the aforementioned topic, an optimal therapeutic approach for epilepsy remains elusive [1]. Therefore, it is critical to investigate new potential treatments that can provide neuroprotective effects and improve seizure symptoms and cognitive function.

In the present study, the researchers investigated the effects of quinpirole, which is a compound known for its potential neuroprotective properties, in an epileptic rat model induced by lithium chloride pilocarpine [2]. Understanding the mechanisms underlying the neuroprotective effects of quinpirole is essential for the development of improved therapeutic strategies for epilepsy.

To evaluate the effects of quinpirole on the symptoms of seizures and cognitive function, an epileptic rat model induced by lithium chloride pilocarpine was utilized. Manifestation of seizures was assessed using the Racine scoring method, and learning and memory functions were evaluated using the Morris water maze test [3]. In addition, electroencephalography (EEG) and immunohistochemistry techniques were used to analyze the protein expression levels of GluR2, BAX, and BCL2 and morphological changes in the hippocampi, which is a key brain region involved in epilepsy [4].

The results of the current study confirmed that the rat model displayed features that were consistent with those of epilepsy. Furthermore, the rats exhibited impaired learning and memory functions, as indicated by the Morris water maze test. The hippocampi of rats in the epileptic group exhibited significantly reduced gene and protein levels of GluR2, suggesting its involvement in the pathogenesis of epilepsy [5].

Quinpirole treatment demonstrated promising results in alleviating symptoms of seizures and improving cognitive function. Notably, quinpirole delayed the onset of seizure and resulted in a decreased frequency of spike waves, as observed by EEG. Moreover, the rats in the quinpirole treatment group exhibited improved performance in the Morris water maze test, with a shorter latency period to reach the platform and an increased number of crossings through the target quadrant.

Furthermore, network pharmacology analysis revealed an intriguing association between quinpirole and GluR2, suggesting its involvement in various signaling pathways associated with epilepsy, such as the cAMP signaling pathway and those related to cocaine addiction and dopaminergic synapses [6–8].

Immunohistochemistry and Western blot analysis further corroborated the neuroprotective effects of quinpirole. Notably, quinpirole treatment resulted in a denser arrangement and more regular morphology of granule cells in the hippocampus, indicating potential structural improvements [9]. Additionally, quinpirole treatment decreased the protein expression of pro-apoptotic BAX and increased the protein expression of anti-apoptotic BCL2 [10.11].

In conclusion, this study demonstrated the neuroprotective effects of quinpirole in a rat model of epilepsy. Improvements were observed in symptoms of seizures and cognitive function, which were associated with the modulation of the protein expression levels of GluR2, BAX, and BCL2. These findings provided valuable insights into the potential therapeutic role of quinpirole in epilepsy treatment and established a foundation for further investigation into the underlying mechanisms. Ultimately, the findings of the current research may contribute to the development of novel treatment strategies for epilepsy.

## Methods

### Animals

Twenty-one-day-old healthy male Wistar rats (weight: 55 ± 3 g) were purchased from the Experimental Animal Center of Shandong University. The rats were randomly divided into the following groups: the control group, the epileptic group, and the quinpirole group. The rats were housed in the animal facility of the Research Centre Laboratory, Tai'an Central Hospital, with a 12-h light/dark cycle and free access to food and water. All the rats were euthanized through an overdose of sodium pentobarbital. Thereafter, they underwent cardiac perfusion with physiological saline for biochemical analysis and were perfused with 4% paraformaldehyde for histological analysis. All the experimental procedures were approved by the Animal Care and Use Committee of the Tai'an Central Hospital and conducted in accordance with the guidelines of the institution. Ethics-related documents are presented in Supplement 1. Lithium chloride (30 mg/kg, Sigma-Aldrich, St. Louis, Missouri, USA) was injected intraperitoneally into the rats, and pilocarpine (30 mg/kg, Sigma-Aldrich) was injected in a similar manner after 24 h. In the control group, an equivalent volume of normal saline was administered as a substitute for pilocarpine. Quinpirole was injected into the lateral ventricle of the rats 60 min before injecting pilocarpine in the quinpirole group. Meanwhile, an equivalent volume of normal saline was injected as a substitute for quinpirole in the epileptic group. After the injection was completed, the syringe was kept at the site of injection for 5 min and then carefully withdrawn. The injection site was 0.7 mm behind the fontanel, 1.3 mm outside the midline, and 3.0 mm in depth [11].

### Evaluation of the symptoms of seizures

The symptoms of seizures were assessed using the Racine scoring method [12], which is a criterion used to determine the level of seizures. Immobile and staring behaviors were defined as grade I, stiff posture was defined as grade II, repeated actions and head tremors were defined as grade III, hind legs standing and myoclonic twitches were defined as grade IV, and rigid clonicity was defined as grade V. For the epileptic model, epilepsy was indicated if a Racine scale of 4–5 and continuous seizures for 30 min were observed. Ninety minutes after the onset of a seizure, a single dose of chloral hydrate (3 ml/kg, Sigma) was administered intraperitoneally to terminate the seizure attack. Further, the duration of seizure incubation and mortality was recorded.

### Morris water maze

The Morris water maze experiment [13] consisted of a spatial acquisition phase (5 days) and an exploratory phase (1 day), which was conducted to assess epilepsy-induced spatial learning impairments in rats. During the spatial acquisition phase, each rat received four training sessions per day from days 1 to 5, with a 20-min interval between each session. In each training session, the rats were expected to find a circular platform with a diameter of 10 cm, which was submerged 2 cm below the water surface in a circular metal pool with a diameter of 180 cm. Once the rats found the platform, it was allowed to stay on it for 10 s. The training time for each trial did not exceed 2 min. The escape latency (in seconds) and the distance to reach the platform (in centimeters) for the rats were recorded using a top-view camera, with the aid of the Noldus Etho Vision XT 10.0 software. In the probe trial, the platform was removed, and the rats were placed on the opposite quadrant for 1 min for the exploration training. The computer tracking system recorded the time spent by the rats in the target quadrant (as a percentage of the time) and the number of crossings.

### Electroencephalography (EEG)

After the induction of anesthesia, the rats were anesthetized using 4% isoflurane in oxygen that was administered through a mask, until they reached the surgical anesthesia plane, which was characterized by the absence of the withdrawal reflex when the toes were pinched. Subsequently, the midline of the scalp was incised and the underlying connective tissue was gently detached to expose the skull. Using sterile surgical instruments, small holes were created at predetermined coordinates to facilitate careful insertion of the electrodes into the brain parenchyma or the cortical surface, thereby minimizing tissue damage. A 16-channel micro-wire array electrode was implanted in the left S1 (anterior–posterior 1.6 mm, medial–lateral 2.0 mm, dorsal–ventral 1.4 mm) for recording purposes. The electrode consisted of a 4 × 4 array of 125-μm 0.5MΩ Pt–Ir electrodes with an inter-electrode spacing of 250 μm. Intraoperative recordings were performed while advancing the 4 × 4 array to confirm the depth of S1. Two miniature screws were secured on the skull above the cerebellum as reference and ground electrodes. Dental acrylic was used to fix all implants onto the bone screws. The animals were allowed one week of recovery before any additional surgeries were performed. After a recovery period of at least 1 h, the rats were placed in a soundproof and temperature-controlled recording chamber. The EEG signals were recorded using a digital EEG amplifier system, with a sampling rate of 50 Hz and a frequency band of 250‒1000 Hz. Before data acquisition, the rats were allowed to adapt to the recording conditions for at least 10 min. The EEG recordings lasted for a minimum of 0.5 h, during which the rats were in a resting awake state. The acquired EEG data were stored for offline analysis, and the raw EEG signals were visually inspected—contaminated segments were excluded after artifact and noise removal, and the remaining data were processed with bandpass filtering and analyzed using the Neuro Explorer software for spectral analysis. The sleep stages were manually scored according to the established criteria, including slow-wave sleep, rapid eye movement sleep, and wakefulness. The statistical analysis of EEG power spectra or event-related potentials was performed using appropriate statistical tests (such as the t-test or repeated measures of analysis of variance).

### Reverse transcription-quantitative polymerase chain reaction (RT‑qPCR)

The primer sequences (Table 1) were synthesized by RIBOBIO (Guangzhou, China). Total RNA from different cell groups was extracted using the TRIzol method (Thermo Fisher Scientific Inc.), and its concentration and purity were measured. The samples were then subjected to PCR amplification using the Eppendorf PCR machine and SYBR® Premix Ex Taq (Accurate Biotechnology Co., Ltd.) for PCR amplification. Real-time quantitative PCR was performed using the Applied Biosystems QuantStudio 5 PCR machine (ABI, Oyster Bay, NY), according to the instructions of the PrimeScript RT Kit with gDNA Eraser (Takara Bio, China, Hunan). The data were analyzed using the 2^−ΔΔCT^ method.

**Table 1:** Primers and their sequences used in quantitative real-time PCR

Gene	Primer-F (5'-3')	Primer-R (5'-3')
*β-actin*	AACAGTCCGCCTAGAAGCAC	CGTTGACATCCGTAAAGACC
*BAX*	CAGGATGCGTCCACCAAGAA	CGTGTCCACGTCAGCAATCA
*BCL2*	TATGATAACCGGGAGATCGTGATC	GTGCAGATGCCGGTTCAGGTACTC
*GluR2*	GCTGGTGGCTTTGATT GAGT	TCTGCGAGGAAGATGGGTTA

### Hematoxylin and eosin staining and immunohistochemistry

The brains were collected and fixed in a 4% formaldehyde solution overnight at 4 °C. The paraffin-embedded sections were dewaxed using xylene, dehydrated with an ethanol gradient, and then stained with HE (Solarbio Science & Technology Co., Ltd., Beijing, China). Five random fields of view were selected on each section for observation under an optical microscope.

Immunohistochemistry techniques were used to analyze the protein expression levels of GluR2, BAX, and BCL2 and morphological changes in the hippocampi of the rats. Paraffin-embedded sections were dewaxed using xylene and dehydrated with an ethanol gradient. The protein expression in the brain tissues was detected using a two-step method (PV-900). The addition of antibody dilution served as the normal control (NC). Subsequently, the sections were washed three times with 0.1 mol/L PBS for 3 min each. Following the washing step, the sections were incubated with 3% peroxidase. Next, the sections were treated with 50 μL of non-immune goat serum for 30 min. Thereafter, the sections were probed overnight at 4 °C with the primary antibody. The primary antibodies used in this study included GLUR2 (1:1000; ab206293; RRID: AB_2800401; Abcam, Massachusetts, USA), anti-Bcl-2 antibody (1:1000; ab196495; RRID: AB_2783814; Abcam, Massachusetts, USA), anti-BAX antibody (1:1000; ab32503; RRID: AB_725631; Abcam, Massachusetts, USA), and anti-β-actin (1:1000; Ta-09; RRID: AB_2636897; ZSGB-BIO, Beijing, China and 1:1000; GB11001; RRID: AB_2801259; Servicebio, Wuhan, China). Additionally, the sections were treated with the polymerase adjuvant at room temperature (25 ± 5 °C) for 20 min and subsequently incubated with the horseradish peroxidase (HRP)-labeled secondary antibody (Beijing Bioss Biotechnology Co., Ltd., Beijing, China) at room temperature for 30 min. The sections were developed using diaminobenzidine (DAB), counterstained with hematoxylin, and mounted for observation. Five random fields of view were selected on each section for observation under an optical microscope.

### Western blot analysis

Western blot analysis was performed to investigate the levels of specific proteins in the cells or tissues subjected to different treatments. Protein extraction was carried out using a radioimmunoprecipitation assay (RIPA) lysis buffer (P0013; Beyotime Biotechnology, Shanghai, China) containing a protease inhibitor (PI) (ST506; Beyotime Biotechnology, Shanghai, China), followed by centrifugation. The resultant supernatant was collected, and its protein concentration was measured using the bicinchoninic acid (BCA) kit (PC0020; Solarbio, Beijing, China). Subsequently, an equal amount of protein was denatured and separated by electrophoresis using 8%, 10%, or 12% SDS-PAGE gels. These proteins were then transferred onto polyvinylidene fluoride membranes (PVDF) (ISEQ00010; Merck KGaA, Darmstadt, Germany). To prevent nonspecific binding, the PVDF membranes were blocked using TBS buffer containing 0.05% tween20 (TBST) and either one of 5% BSA or defatted milk. Next, the membranes were incubated overnight at 4 °C with the primary antibodies diluted in TBST, with either 5% BSA or defatted milk, according to the manufacturer's instructions. After three washes with TBST, the membranes were incubated with the secondary antibodies for 1 h at room temperature. Finally, the immunoblots were visualized using an ECL kit (WBKLS0100; Millipore, Massachusetts, USA).

### Construction of protein–protein interaction (PPI) networks

To construct protein–protein interaction (PPI) networks, established PPI databases such as STRING (https://string-db.org/) and PINA (https://omics.bjcancer.org/pina/) were used. These databases provide a collection of extensive protein–protein interaction data as well as relevant algorithms and tools for constructing PPI networks. Utilizing this interaction data, a model of the protein–protein interaction network was established, where the proteins were denoted as nodes and the interactions as edges. Subsequently, various methods such as node degree centrality, module analysis, and network clustering were employed to analyze the constructed PPI networks. These methods enable the identification of characteristics and functional modules within the protein–protein interaction networks. In addition, pathway estimation analysis was performed on the core proteins using the KEGG database (https://www.kegg.jp/), and the outcomes were visualized using R software.

### Statistical analysis

Image J was used to analyze the grayscale values of Western blot and immunohistochemistry. For this, the IHC toolbox software package was used. The data were analyzed using appropriate statistical tests. Specifically, the t-test was employed for comparing two groups, whereas one-way analysis of variance (ANOVA) was employed for comparing multiple groups. A *P*-value < 0.05 was considered statistically significant. GraphPad Prism 8 was utilized for creating statistical graphs.

## Results

### Confirmation of epileptic features

**Fig. 1:**
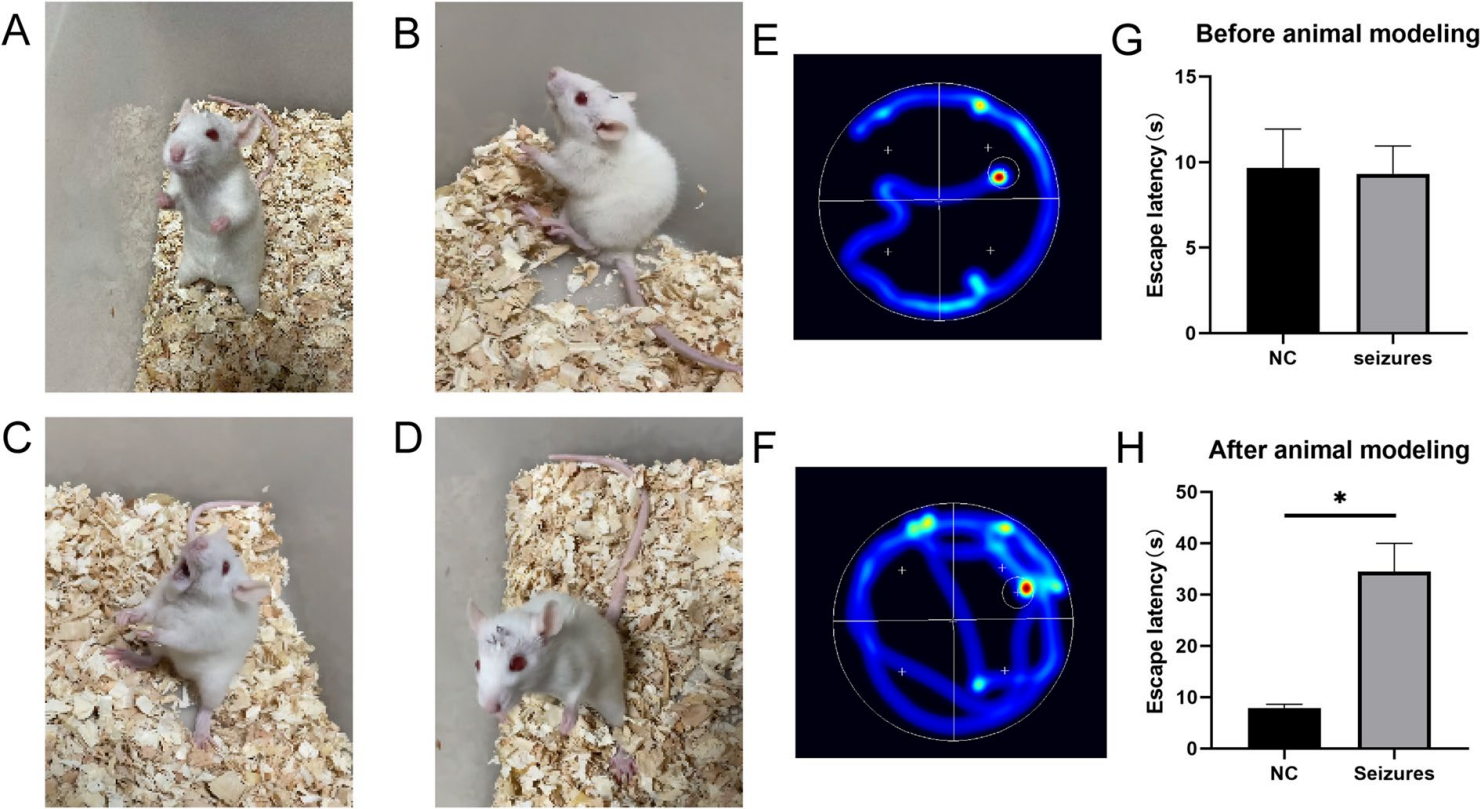
The seizure group exhibited typical features of a seizure. The water maze confirmed the presence of learning and memory impairments in the seizure group. **A**-**D** The seizure group exhibiting typical features of a seizure. **E** NC group in the water maze. **F** Seizure group in the water maze. **G** Statistical analysis of escape latency before modeling. **H** Statistical analysis of escape latency after modeling. Each group *N* = 3, *: *P* < 0.05

As compared to the control group, the rats in the epileptic group exhibited typical symptoms of epilepsy, with Racine scores of IV–V (Fig. 1A–D). In the water maze test, the rats in the epileptic group demonstrated significantly lower time spent on the platform and fewer crossings of the target quadrant as compared to the control group, indicating significantly impaired learning and memory functions in the epileptic group (Fig. 1E–H).

### Changes in GLUR2 expression

**Fig. 2:**
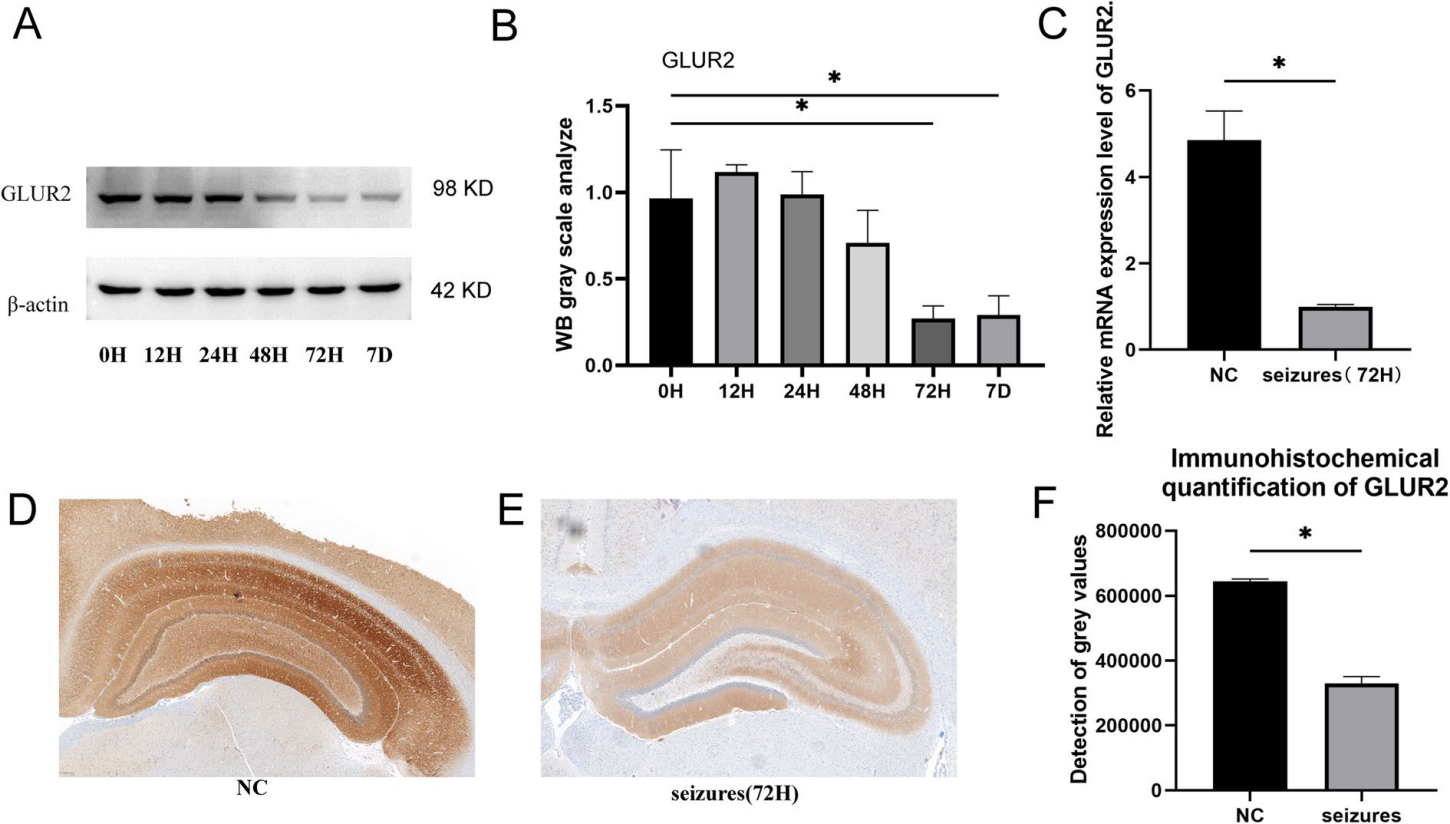
After 72 h of seizure occurrence, the expression of GluR2 significantly decreases in the hippocampus. **A** Western blot analysis showed that the protein expression of GluR2 in the hippocampi of rats in the seizure group was significantly downregulated 72 h after the occurrence of the seizure. **B** Quantitative Western blot analysis results for GLUR2 protein. **C** PCR confirmed that the expression of the GluR2 gene in the hippocampi of rats in the seizure group was significantly downregulated 72 h after the occurrence of the seizure. **D** Immunohistochemical analysis of GLUR2 in the NC group. **E** Immunohistochemical analysis of GLUR2 in the seizures (72H) group. **F** Quantitative results of immunohistochemistry between the two groups. Each group *N* = 3, *: *P* < 0.05

After 72 h of the episode of seizure, the most significant decrease was observed in GLUR2 levels. The results of the Western blot indicated that after epilepsy occurred, the expression level of GLUR2 consistently showed a decreasing trend, with the most significant decrease observed after 72 h of the seizure (Fig. 2A, B). The outcomes of the RT-PCR analysis were consistent with the aforementioned findings (Fig. 2C). A significant decrease in the gene expression level of GLUR2 was observed after the occurrence of epilepsy (*P* < 0.05). The results obtained from immunohistochemistry were highly consistent with those from Western blot (Fig. 2D–F).

### Effects of quinpirole on symptoms and cognitive function

**Fig. 3:**
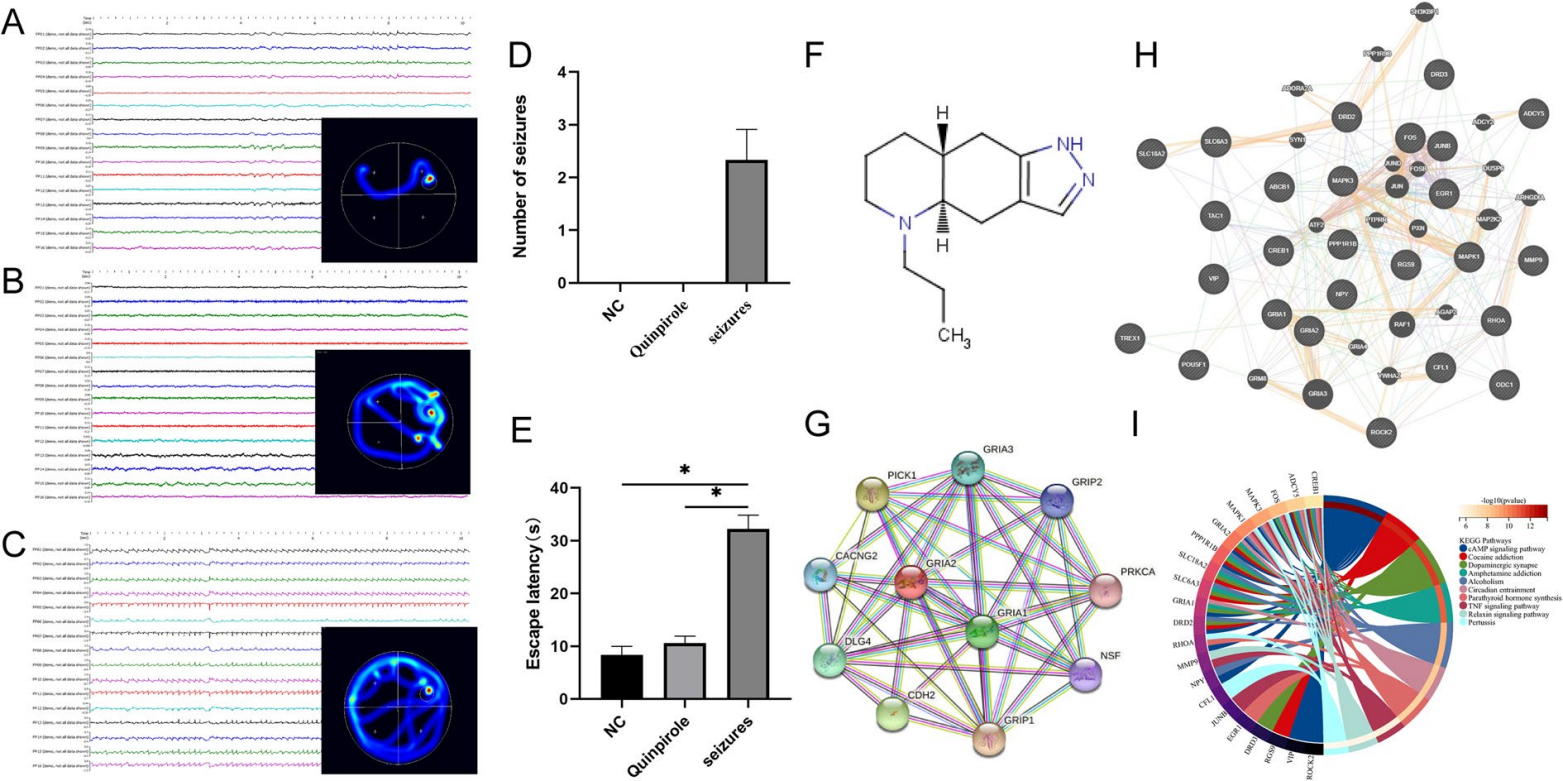
Electroencephalogram and water maze results after the action of quinpirole in an epilepsy model and its network pharmacology analysis. **A** Electroencephalogram and water maze results in the NC group. **B** Electroencephalogram and water maze results in the quinpirole group. **C** Electroencephalogram and water maze results in the seizures group. **D** The occurrence of epileptic seizures was recorded during the electroencephalogram (EEG) monitoring period. **E** The comparison of water maze results among the three groups. **F** Molecular structure diagram of quinpirole. **G** STRING database of proteins that interact with GRIA2. **H** Protein Interaction Network Analysis database of proteins that interact with GRIA2. **I** KEGG analysis of proteins interacting with GRIA2. *N* = 3 in each group, *: *P* < 0.05

The seizure incubation period of rats in the quinpirole group was significantly longer than that in the epileptic group. EEG analysis showed a significant decrease in the frequency of spike waves in the quinpirole group than that in the epileptic group. In the Morris water maze test, the quinpirole group had a significantly shorter latency period to reach the hidden platform and demonstrated an increased number of crossings through the target quadrant, indicating improved learning and memory functions (Fig. 3A–E). Quinpirole is an organic compound whose molecular structure consists of a repeating ring structure. The molecule contains a nitrogen atom (N) attached to two carbon atoms (C) and a chlorine atom (Cl). These two carbon atoms form a longer carbon chain, with one end of the chain attached to another carbon atom and the other end attached to the ring structure. The carbon atom in the ring structure is connected to the nitrogen atom, thereby forming a five-membered ring. In other parts of the molecule, some hydrogen atoms (H) also attach to the carbon atoms. This molecular structure imparts quinpirole its specific chemical properties and pharmacological activity (Fig. 3F). The STRING database showed that GIRA2 interacted with other proteins, including GIRA1 and GIRA3, among 10 proteins (Fig. 3G). This suggested that in the cells, GIRA2 may interact with GIRA1 and GIRA3 proteins, thereby participating in common biological processes or functions. The discovery of these protein–protein interactions can provide clues for further research, revealing an interaction network and signaling pathways of these proteins within the cells. The Protein Interaction Network Analysis (PINA) database showed that GIRA2 interacted with even more proteins (Fig. 3H). Further, KEGG analysis indicated that quinpirole may exert its effects after epilepsy through the cAMP signaling pathway and other pathways related to cocaine addiction and dopaminergic synapses (Fig. 3I).

### Expression levels and morphological changes in GluR2, BAX, and BCL2 proteins

**Fig. 4:**
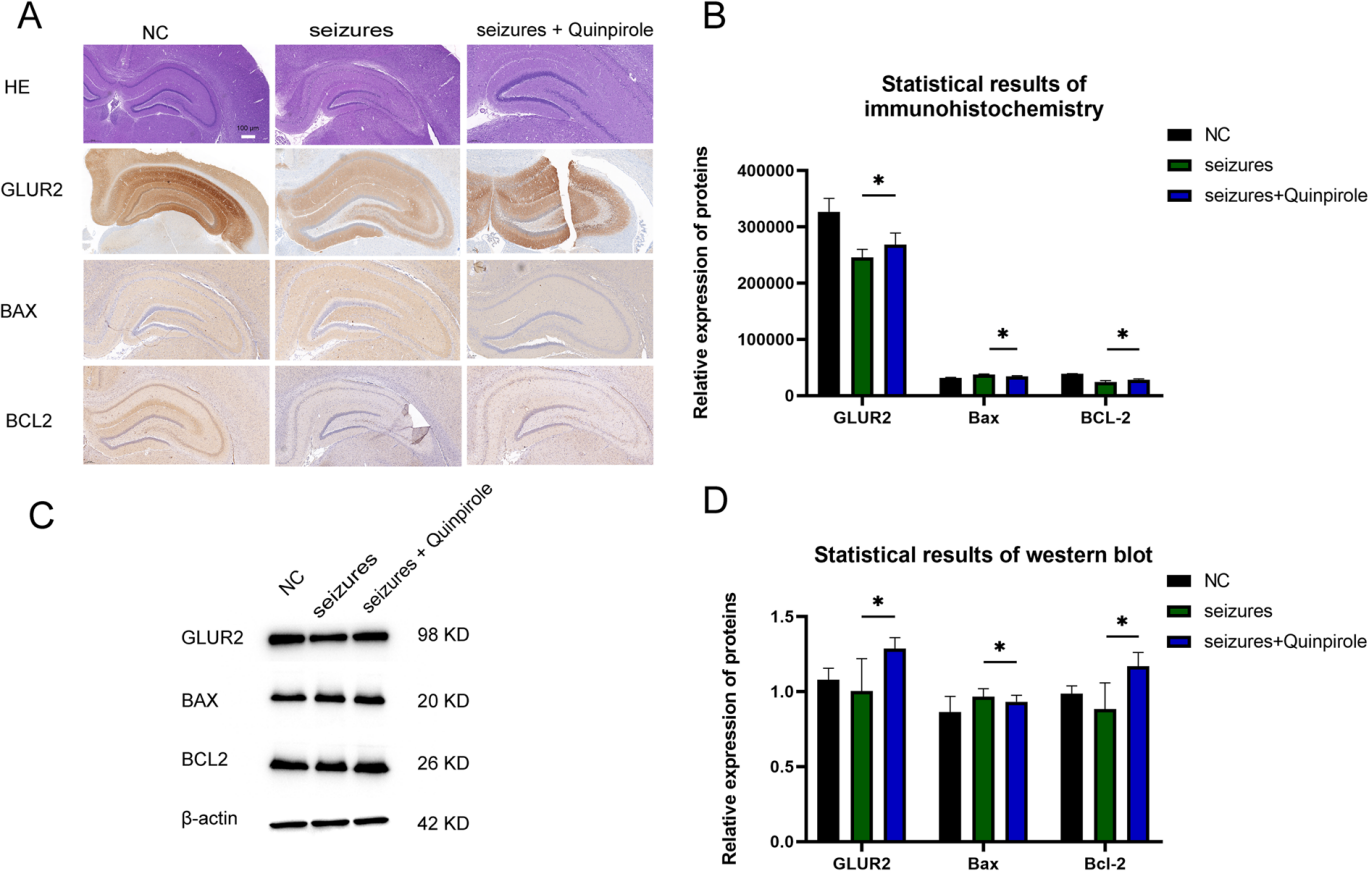
Pathologic changes and apoptosis-related proteins after the action of quinpirole in a seizure model. **A** Hematoxylin and eosin staining and immunohistochemical staining after the action of quinpirole in a model of epilepsy. **B** Quantitative immunohistochemistry results. **C** Quinpirole acts on GLUR2 and apoptosis-related proteins in the epileptic group as detected by Western blot. **D** Quantitative results of Western blot analysis. Each group *N* = 3, *: *P* < 0.05

Notably, the cells in the dentate gyrus region of the rat hippocampus in the epileptic group were looser and more irregularly shaped as compared to those in the control group. Further, the protein expression of GLUR2 and BCL2 was reduced, whereas the protein expression of BAX was increased. However, after quinpirole treatment, the granule cells in the rat hippocampus were more densely arranged and had a more regular shape. Furthermore, the protein expression of GLUR2 and BCL2 was increased, whereas the protein expression of BAX was decreased (Fig. 4A, B). The results from the Western blot analysis were consistent with those from the immunohistochemical analysis (Fig. 4C, D).

## Discussion

Epilepsy is a complex neurological disorder characterized by recurrent seizures, and it can exert significant detrimental effects on a patient’s quality of life [14]. Although several treatments are available for epilepsy, such as surgery, vagus nerve stimulation, dietary therapy, and optogenetic techniques in addition to medications, they are often insufficient in controlling seizures and preventing cognitive decline [15–17]. Therefore, new therapeutic approaches that can effectively manage seizures and protect the brain from further damage need to be explored.

In the current study, the potential neuroprotective effects of quinpirole were investigated in an epileptic model induced by lithium chloride pilocarpine. Quinpirole is a dopamine receptor agonist that reportedly exerts various effects on the central nervous system [18, 19]. Previous studies have suggested the neuroprotective properties of quinpirole in different neurological disorders, such as Parkinson's disease and ischemic stroke [20, 21]. However, its potential therapeutic effects in an epileptic model induced by lithium chloride pilocarpine have not been explored extensively. The results of the current study demonstrated that quinpirole treatment significantly delayed the onset of seizures and decreased the mortality rate after the induction of seizures in the epileptic model induced by lithium chloride pilocarpine. This suggested that quinpirole may have antiepileptic properties and that it could be a potential treatment option for controlling seizures in patients with epilepsy. These findings were consistent with those of previous studies, which demonstrated the effectiveness of dopamine receptor agonists in reducing seizure activity [22, 23]. The cognitive function of rats with epilepsy was also assessed using the Morris water maze test. The epileptic group exhibited significant impairments in learning and memory, which was consistent with cognitive deficits observed in patients with epilepsy [24, 25]. However, rats treated with quinpirole showed improved cognitive performance, as indicated by a reduced latency period to reach the platform and an increased number of crossings of the target quadrant. These results suggested that quinpirole treatment not only reduced seizures, but also preserved cognitive function in rats with epilepsy induced by lithium chloride pilocarpine. Frequent and sustained seizures may cause changes in the variety of amino acids and neurotransmitters in the brain, further resulting in neuronal necrosis in the responding regions [26]. To further understand the underlying mechanisms of the neuroprotective effects of quinpirole, the protein expression levels of GluR2, BAX, and BCL2 in the hippocampus were investigated. GluR2 is a subunit of the AMPA receptor, which plays a critical role in excitatory synaptic transmission [27]. In the current study, a significant decrease in GluR2 expression was observed in the hippocampi of rats with epilepsy. This reduction in GluR2 expression may contribute to the increased permeability for calcium ions and apoptosis of neuronal cells observed in epilepsy [28]. Interestingly, the quinpirole treatment reversed the decrease in GluR2 expression, suggesting that it may regulate glutamate signaling and restore the balance between excitatory and inhibitory neurotransmission [10, 29, 30]. This was consistent with the findings of previous studies, which demonstrated the neuroprotective effects of dopamine receptor agonists on glutamatergic neurotransmission [31].

Moreover, network pharmacology analysis revealed a close relationship between quinpirole and GluR2, further supporting the involvement of glutamate signaling in the therapeutic effects of quinpirole. The decreased expression of GluR2 in a variety of neurological diseases increases the inward flow of calcium ions, which can activate protease, phospholipase, and ATPase, ultimately leading to cellular swelling and apoptosis of neurocytes. Additionally, KEGG pathway analysis suggested the potential involvement of the cAMP signaling pathway and other pathways related to cocaine addiction and dopaminergic synapses [32–34]. Furthermore, the protein expression levels of BAX and BCL2 were investigated, which are involved in regulating apoptosis. In the epileptic group, an increase in BAX expression and a decrease in BCL2 expression were observed, indicating an imbalance between pro-apoptotic and anti-apoptotic factors. However, quinpirole treatment reversed these changes, suggesting its potential role in inhibiting apoptosis and promoting neuronal survival [35, 36].

In conclusion, the study highlights promising neuroprotective effects of quinpirole in an epileptic model induced by lithium chloride pilocarpine. Further, quinpirole treatment effectively reduced seizure activity, improved cognitive function, and regulated the protein expression of GLUR2, BAX, and BCL2 in the hippocampus. These findings provided important insights into the potential therapeutic benefits of quinpirole and its underlying mechanisms in the treatment of epilepsy. Further studies are warranted to explore the clinical potential of quinpirole and its optimal dosage and treatment regimen for epilepsy.

## Data Availability

Not applicable.
